# Improving Completion of a Routine Outcome Measure at Admission and Discharge in a National Health Service Crisis Team: A Quality Improvement Study

**DOI:** 10.7759/cureus.105379

**Published:** 2026-03-17

**Authors:** Usman A Abdul-Quayum

**Affiliations:** 1 Mental Health, University of Glasgow, Glasgow, GBR; 2 Psychiatry, Dykebar Hospital, Glasgow, GBR

**Keywords:** audit, core-10, crisis services, mental health, quality improvement, routine outcome monitoring, service evaluation

## Abstract

Background

Routine outcome monitoring is an important component of contemporary mental health care. Completion of structured outcome measures at admission and discharge supports clinical decision-making and service evaluation. Local policy within National Health Service Greater Glasgow and Clyde (NHS Greater Glasgow and Clyde) requires completion of the Clinical Outcomes in Routine Evaluation - 10 item measure (CORE-10) at these time points. This study evaluated baseline compliance within the Renfrewshire Intensive Home Treatment Team (IHTT) and assessed the impact of targeted quality improvement interventions.

Methods

A quality improvement audit cycle study was conducted across two audit cycles in August and November 2025. All patients admitted by the service during each month were included. The primary outcome was documented completion of the CORE-10 measure at admission and discharge. Interventions implemented between cycles included structured teaching sessions, visual reminders, and weekly multidisciplinary team prompts. Proportions were compared using chi-square testing.

Results

Twenty-nine patients were included in cycle one and twenty-eight in cycle two. Admission completion improved from four of twenty-nine patients (13.8%) to fifteen of twenty-eight patients (53.6%) following intervention (χ² = 10.04, p = 0.002). Discharge completion remained low, from one of twenty-nine patients (3.4%) to one of twenty-eight patients (3.6%) (χ² = 0.00, p = 0.97).

Conclusion

Targeted educational and team-based interventions significantly improved admission completion of a routine outcome measure within a crisis service. However, discharge completion remained poor, suggesting persistent structural barriers at transition points in care. Further system-level strategies are required to embed sustainable discharge outcome monitoring.

## Introduction

Routine outcome monitoring has become an established component of modern mental health care. Standardised self-report measures allow quantification of psychological distress, facilitate shared clinical decision-making, and support service evaluation. Evidence suggests that when feedback from outcome measures is systematically incorporated into clinical practice, it may improve therapeutic engagement and support more responsive treatment planning, although effects vary across settings [1,2].

The Clinical Outcomes in Routine Evaluation - 10 item measure (CORE-10) is a brief, validated, ten-item instrument derived from the longer Clinical Outcomes in Routine Evaluation - Outcome Measure (CORE-OM) and is widely used across United Kingdom mental health services [3-5]. Its brevity and psychometric robustness make it suitable for routine use across a range of service settings, including community and crisis services.

Crisis resolution and home treatment services operate in high-acuity environments characterised by rapid patient turnover, short duration of care episodes, and frequent multidisciplinary handovers. These operational pressures may create structural barriers to consistent documentation of outcome measures, particularly at discharge when clinical focus shifts toward risk management and onward care planning. Despite national professional guidance emphasising the importance of systematic and standardised outcome measurement to improve quality and transparency in mental health services [6], variability in compliance persists.

Within the National Health Service Greater Glasgow and Clyde (NHS Greater Glasgow and Clyde), local policy and standard operating procedures require completion of the CORE-10 measure at admission and discharge within community and crisis services. The Renfrewshire Intensive Home Treatment Team provides short-term, community-based crisis intervention bridging outpatient and inpatient psychiatric care, and therefore represents a relevant setting in which to examine adherence to routine outcome documentation standards.

This study aimed to evaluate the implementation of routine outcome monitoring within a crisis resolution service. The objectives were twofold: (1) to assess baseline compliance with completion of the CORE-10 measure at admission and discharge, and (2) to evaluate whether targeted quality improvement interventions implemented between audit cycles improved completion rates.

## Materials and methods

Study design and setting

This study was conducted within the Renfrewshire Intensive Home Treatment Team (IHTT), part of NHS Greater Glasgow and Clyde, based at Dykebar Hospital in Glasgow, Scotland. The IHTT provides short-term crisis intervention for adults experiencing acute mental health deterioration as an alternative to inpatient admission. A quality improvement audit cycle methodology was used to assess completion of the CORE-10 measure at both admission and discharge. The project consisted of two retrospective data collection periods separated by the implementation of a prospective quality improvement intervention aimed at improving compliance with routine outcome monitoring.

The CORE-10 is a brief self-report measure of psychological distress derived from the longer CORE-OM [3,4]. It consists of ten items scored on a five-point Likert scale (0-4), yielding a total score ranging from 0 to 40, with higher scores indicating greater psychological distress. The CORE-10 user manual provides guidance on scoring thresholds and clinical interpretation [5].

Sample and data collection

Two audit cycles were conducted over the entire months of August 2025 and November 2025. All patients accepted by the Renfrewshire IHTT during each audit month were included. As the audit aimed to capture all eligible admissions during the specified periods, no sampling was performed, and the full cohort of patients was analysed. Inclusion criteria were adults aged eighteen years or older accepted for crisis intervention during the specified months. Exclusion criteria were patients transferred directly to inpatient care before assessment or those with incomplete electronic records, preventing confirmation of CORE-10 documentation status. Electronic clinical records were reviewed to determine whether the CORE-10 measure had been completed at admission and discharge. Where completed, admission and discharge scores were recorded. As this was a service evaluation audit of all eligible cases during predefined timeframes, formal sample size estimation was not undertaken. The project aimed to capture the total population of service users within each audit period.

Interventions

Between audit cycles, a structured quality improvement intervention was implemented. This included two structured teaching sessions delivered during protected team education time, focusing on the rationale for routine outcome monitoring, correct administration of the CORE-10, and interpretation of scores. Visual reminder posters were displayed in clinical work areas, summarising when the CORE-10 should be completed (at admission and discharge), scoring thresholds (0-5 healthy, 6-10 low level problems, 11-20 mild, 21-25 moderate, ≥26 severe), and documentation location within the electronic record. In addition, weekly multidisciplinary team (MDT) prompts were incorporated into team handovers. During these discussions, the presence or absence of CORE-10 documentation for newly admitted and recently discharged patients was verbally confirmed. These reminders were delivered verbally rather than through a formal checklist. Lastly, feedback from the baseline audit was shared with staff, and barriers to completion were discussed. 

**Figure 1:**
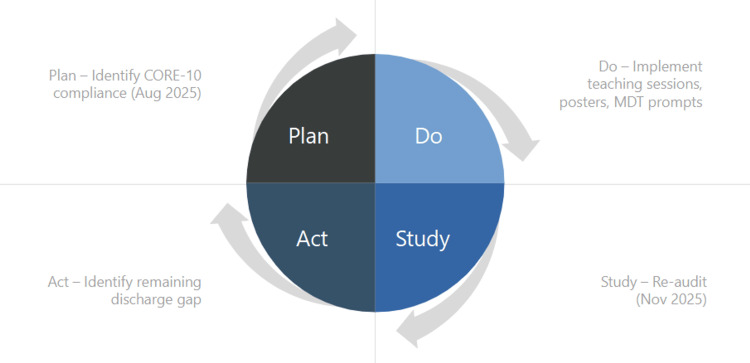
Plan-Do-Study-Act (PDSA) cycle applied to improve CORE-10 completion Illustrative circular representation of the PDSA cycle used in this quality improvement project, demonstrating baseline audit (August 2025), implementation of interventions, re-audit (November 2025), and identification of ongoing discharge documentation gaps. CORE-10: Clinical Outcomes in Routine Evaluation - 10-item measure; MDT: multidisciplinary team

The intervention was implemented using the Plan-Do-Study-Act (PDSA) quality improvement framework. During the planning phase, baseline audit findings were reviewed, and barriers to completion were identified. The intervention phase involved implementation of staff education, visual prompts, and MDT reminders. In the study phase, compliance with CORE-10 completion was reassessed during the second audit cycle. The act phase involved reviewing findings and identifying further opportunities to improve documentation practices. The overall Plan-Do-Study-Act (PDSA) cycle is illustrated in Figure 1.

Outcome measures

The primary outcome was documented completion of the CORE-10 measure at admission and at discharge. Compliance was defined as the presence of a completed measure within the electronic record at the relevant time point.

Ethical considerations

This project was registered locally as a quality improvement initiative within the National Health Service Greater Glasgow and Clyde, and did not require formal ethical approval. The CORE-10 instrument was not reproduced within this manuscript, and no copyrighted material was included.

Statistical analysis

Statistical analysis was performed using IBM SPSS Statistics for Windows, Version 29.0 (IBM Corp., Armonk, USA). Proportions of completed measures were calculated for each audit cycle. Categorical variables were compared using the chi-square (χ²) test of independence. Differences in completion rates between audit cycles were compared using the chi-square (χ²) test of independence. Statistical significance was defined as p < 0.05.

## Results

**Figure 2:**
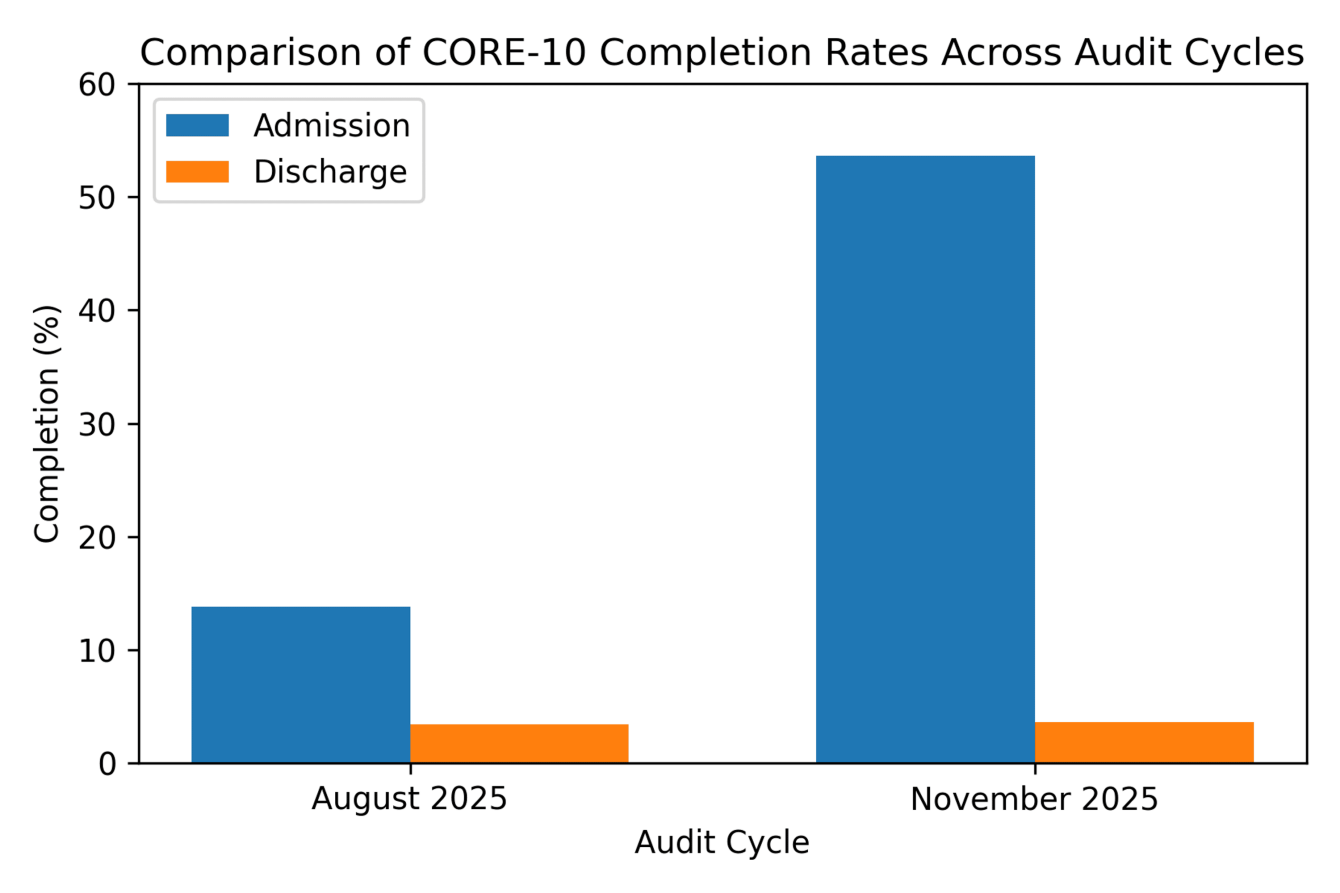
Comparison of CORE-10 completion rates across audit cycles Bar chart showing percentage completion of the CORE-10 at admission and discharge during cycle one (August 2025) and cycle two (November 2025). CORE-10: Clinical Outcomes in Routine Evaluation - 10-item measure

Twenty-nine patients were included in cycle one and twenty-eight patients in cycle two. Completion rates across audit cycles are presented in Table 1 and illustrated in Figure 2.

**Table 1:** Completion of routine outcome measures across audit cycles Completion of routine outcome measurement across audit cycles. Values represent original data collected from the Renfrewshire Intensive Home Treatment Team (IHTT) (NHS Greater Glasgow and Clyde). Data are presented as number (n) and percentage (%) of patients with documented completion. Statistical comparison between cycles was performed using the chi-square (χ²) test of independence. This project was registered locally as a quality improvement initiative.

Audit cycle	Total patients (n)	Admission completion n (%)	Discharge completion n (%)	p-value
August 2025	29	4 (13.8%)	1 (3.4%)	–
November 2025	28	15 (53.6%)	1 (3.6%)	Admission: 0.002; Discharge: 0.97

Admission completion

In cycle one, four of twenty-nine patients (13.8%) had a completed measure at admission. In cycle two, fifteen of twenty-eight patients (53.6%) had a completed measure. This represented a statistically significant improvement following intervention (χ² = 10.04, p = 0.002).

Discharge completion

In cycle one, one of twenty-nine patients (3.4%) had a completed measure at discharge. In cycle two, one of twenty-eight patients (3.6%) had a completed measure. There was no statistically significant difference between cycles (χ² = 0.00, p = 0.97).

Given the limited number of completed discharge measures, comparison of admission and discharge CORE-10 scores was not undertaken.

## Discussion

This quality improvement study demonstrated a substantial and statistically significant improvement in admission completion of a routine outcome measure following targeted educational and team-based interventions within a crisis service. Admission compliance increased from 13.8% to 53.6% across audit cycles, indicating that relatively simple interventions can influence documentation practices in high-acuity settings.

The CORE-10 is a brief, psychometrically validated instrument derived from the longer CORE-OM [3,4]. It demonstrates good internal consistency (Cronbach’s alpha typically >0.85), strong convergent validity with measures of depression and anxiety, and sensitivity to clinical change across short treatment intervals [3,4]. Its brevity and ease of scoring make it particularly suitable for high-acuity crisis services, where time constraints and rapid patient turnover may limit the feasibility of longer outcome instruments. These properties support its use as a pragmatic routine outcome monitoring tool within intensive community mental health teams.

Routine outcome monitoring is associated with improved clinical feedback, enhanced patient engagement, and greater service accountability [2,6]. In this study, structured teaching, visual reminders, and repeated multidisciplinary prompts likely increased awareness and normalised expectations regarding completion at admission. Sharing baseline audit results also promoted staff engagement and accountability.

Despite improvement at admission, discharge completion remained extremely low. This suggests persistent structural and workflow barriers at transition points in care. Discharges in crisis services may occur rapidly and under time pressure, limiting opportunities for formal outcome measurement. Competing clinical priorities during discharge planning, including risk assessment and care coordination, may further reduce the likelihood that outcome measures are completed. The absence of embedded electronic prompts or standardised discharge templates may also contribute to non-compliance.

These findings are consistent with previous quality improvement literature, indicating that educational interventions alone may be insufficient to achieve sustained change in complex clinical environments [7,8]. Future improvement cycles should consider system-level interventions, such as mandatory electronic documentation fields or integrated discharge checklists.

Limitations

This study was conducted within a single service over two discrete months, limiting generalisability. Sample sizes were modest, reflecting the scale of the service. The project focused on process measures rather than clinical outcomes. In addition, the analysis examined documentation of CORE-10 completion rather than individual domain scores within the instrument (problems, functioning, and risk). As a quality improvement initiative, unmeasured contextual factors may have influenced results. The absence of qualitative staff feedback also limits the interpretation of contextual barriers.

Additionally, the potential for Hawthorne Effect bias must be acknowledged. Staff awareness of being audited may have temporarily improved documentation behaviour, independent of sustained cultural change. Longer-term follow-up would be required to determine the durability of the observed improvements.

Future directions

Future work should evaluate system-based modifications to embed discharge completion within routine workflows. Further cycles may also assess whether improved outcome monitoring influences clinical decision-making and patient outcomes.

## Conclusions

Targeted educational and team-based interventions significantly improved completion of a routine outcome measure at admission within a National Health Service crisis team. However, discharge completion remained persistently low, highlighting the need for structural and workflow-based solutions at care transition points. Embedding outcome monitoring into routine crisis service practice requires not only staff awareness but systematic integration into admission and discharge processes to ensure sustainable compliance and support high-quality patient care.
